# A Community-Based Lifestyle-Integrated Physical Activity Intervention to Enhance Physical Activity, Positive Family Communication, and Perceived Health in Deprived Families: A Cluster Randomized Controlled Trial

**DOI:** 10.3389/fpubh.2020.00434

**Published:** 2020-09-15

**Authors:** Agnes Y. K. Lai, Eliza Y. W. Lam, Cecilia Fabrizo, Dickson P. K. Lee, Alice N. T. Wan, Jessica S. Y. Tsang, Lai-ming Ho, Sunita M. Stewart, Tai-hing Lam

**Affiliations:** ^1^School of Nursing, The University of Hong Kong, Hong Kong, Hong Kong; ^2^Caritas-Hong Kong, Hong Kong, Hong Kong; ^3^School of Public Health, The University of Hong Kong, Hong Kong, Hong Kong; ^4^Department of Psychiatry, The University of Texas Southwestern Medical Center, Dallas, TX, United States

**Keywords:** community-based, theory-based, physical activity, Zero-time exercise, positive family communication

## Abstract

**Background:** Zero-time exercise (ZTEx) is an approach integrating simple strength- and stamina-enhancing physical activity into daily life. The study evaluated the effectiveness of a community-based lifestyle-integrated physical activity intervention using ZTEx to enhance participants' physical activity, family communication, perceived health and happiness, and family harmony.

**Methods:** A parallel group, cluster randomized controlled trial was conducted in a sample of 673 participants from eight Integrated Family Service Centers in Hong Kong. The experimental group (*n* = 316) received a physical activity intervention. The control group (*n* = 357) received information on healthy eating. Both groups received three face-to-face intervention sessions (totalling 6 h and 30 min) and 16 text messages. The primary outcome was the change in days spent engaged in ZTEx. Secondary outcomes included changes in sitting time, days engaged in moderate or vigorous physical activities, family communication (encouraging and engaging family members in ZTEx), dietary habits, perceived health and happiness, and family harmony. Self-administered questionnaires were used at baseline and at 3, 6, and 12 months. Mixed effects models with intention-to-treat analysis was used.

**Results:** Compared with the control group at 3 months, the experimental group showed significantly greater increases of 1.3 days spent doing ZTEx (Cohen's *d*: 0.60), 0.3 days spent doing moderate physical activity (Cohen's *d*: 0.08), 0.3 days encouraging family members to do ZTEx (Cohen's *d*: 0.16), and 0.7 days doing ZTEx with family members (Cohen's *d*: 0.39) during the 7 days prior. At 3 months, the experimental group also showed a significantly greater improvement in perceived health, by a score of 0.2 (Cohen's *d*: 0.14). The effect sizes ranged from small to medium, with similar intervention effects at the 6-month and 1-year assessments. Compared with the experimental group, the control group showed a significantly greater reduction of 0.4 days on which sweetened beverages were consumed (95% CI: 0.01, 0.9, *p* < 0.05, Cohen's *d*: 0.28). The qualitative results supported the quantitative findings.

**Conclusions:** Our findings show that a community-based lifestyle-integrated physical activity (PA) intervention can enhance physical activity, family communication, and perceived health in deprived families in Hong Kong.

**Trial registration:** The research protocol was retrospectively registered at the National Institutes of Health (identifier number: NCT02601534) on November 10, 2015.

## Introduction

Physical activity has significant positive health effects on all age groups (1). However, a large proportion of the global population (28% of adults aged 18 years or more and more than 80% of school-going adolescents aged 11–17 years) have inadequate levels of physical activity and a sedentary lifestyle (2). Studies have consistently demonstrated that a sedentary lifestyle can contribute to obesity, type 2 diabetes, some types of cancer, cardiovascular diseases, and early death (3). Adults are essential role models for their children, and the reciprocal nature of the adult–child relationship influences the health behavior of both children and adults (4). Exercising with family members has been recognized as the most rewarding activity to benefit one's well-being (5).

Community-based interventions have the potential to achieve population-level impact as they reach target groups in their natural environment (6). The School of Public Health of the University of Hong Kong (HKU-SPH), in collaboration with Caritas–Hong Kong, conducted a community-based intervention entitled “Effective Parenting Programme” to ease parents' frustrations in parenting young children (7). Caritas–Hong Kong is a charitable non-governmental organization focused on care and support for deprived families, including single parents, new arrivals, and low-income families. Deprived families report multiple health problems and lower ratings on happiness scales, grapple with more family problems and are more likely to pay less attention to their well-being than the general population (8). Building on established academic and community relationships (7), HKU-SPH collaborated with Caritas-HK to launch another community-based project entitled “Family Education Project” (FEP) for deprived families to enhance perceived well-being through promoting physical activity and doing exercise with family members.

Most reports of community-based physical activity interventions involving family members in the extant literature have been based in Western countries, such as the United States (9, 10), Canada (11–13), Australia (14, 15) and the United Kingdom (16). These interventions focused on outdoor activities, which may not be as easily applicable to a city like Hong Kong due to environmental, social, and cultural differences (17). Hong Kong is a space-limited, densely populated city with about 95% Chinese, where most parents and children tend to focus on their children's academic performance instead of physical activity levels (18). The majority of people are preoccupied with their daily lives, pay less attention to physical activity or family time, and have the belief that regular exercise is time-consuming and expensive (18, 19). Currently, existing reports of community-based physical activity interventions for Chinese communities target weight control in either overweight/obese adults or children, but not preventive work for the general public (20, 21).

To overcome these barriers, HKU-SPH created “Zero-time exercise” (ZTEx), a new approach to kick-start the integration of simple strength- and stamina-enhancing physical activity, such as simple movements and stretching while sitting or standing, into daily life. ZTEx does not require extra time, money, and equipment, and can be done anytime, anywhere and by anybody (22). ZTEx uses a foot-in-the-door approach to start exercise in small steps, building exercise self-efficacy. This approach is in line with the suggestion from physical activity guidelines for Americans that moving more and sitting less will benefit nearly everyone, and some physical activity is better than none (23). ZTEx can also be an innovative creative fun family activity (e.g., family members of all ages can create and participate in friendly competition games) (24). Examples of ZTEx while sitting and standing include pedaling both legs and standing on one leg, respectively. More examples of ZTEx are shown in our YouTube videos (https://www.youtube.com/user/familyhk3h/videos). Our pilot trials on ZTEx for lay health promoters (*n* = 28), social service and related workers (*n* = 56), individuals with insomnia (*n* = 37) and the elderly (*n* = 151) showed increased physical activity and perceived well-being (22–27).

The current study extended the findings on ZTEx from pilot trials to a large-scale cluster randomized controlled trial (cRCT). Our physical activity (PA) intervention emphasized that ZTEx is easy, enjoyable, and effective and aimed to enhance participants' physical activity, family communication, and perceived well-being. Grounded on components of the Health Action Process Approach for behavioral change (28), the PA intervention and design of text messages targeted cognitive factors for the formation of exercise motivation (e.g., risk perception, exercise self-efficacy, and outcome expectations) and regulatory factors for regular physical activity (e.g., exercise goal-setting and planning, and self-monitoring). Over time, action control was expected to develop and become a habit. This approach is in line with Rhodes's multi-process action control approach for physical activity behavior (29). In addition, we extended the traditional exercise promotion model, which emphasizes service delivery to others, by harnessing the opportunity to treat parents as role models for their family members. Role modeling is a powerful teaching strategy (30), and the approach has been applied to enhance positive health behaviors (such as physical activity) in children (31) and adolescents (32).

We hypothesized that (i) participants in the experimental group would display significantly greater increases in simple strength- and stamina-enhancing physical activity (i.e., ZTEx), physical activity, and family communication through encouraging and engaging family members in ZTEx, as well as improvements in perceived health and happiness, and family harmony; and (ii) the family members of participants in the experimental group would be more physically active than those in the control group. This paper reports the development, feasibility, and preliminary evidence on the effectiveness of the PA intervention.

## Methods

### Design

A cRCT randomized eight Caritas–Hong Kong Integrated Family Service Centers (IFSCs) into the “PA intervention” experimental group or the “healthy eating” control group at a 1:1 ratio by creating a random sample in Microsoft Excel. The randomization sequence was generated by a research staff who was not involved in the recruitment process, intervention, or data collection. Both groups comprised four IFSCs. Each IFSC conducted two to three classes (20–40 participants per class) on different days of the same week with identical content and duration of sessions. This arrangement enabled participants to choose the most convenient day of the week for them to attend.

### Participants

The FEP was publicized in all participating IFSCs. Individuals who were interested in the FEP were recruited if they fulfilled the following inclusion criteria: (i) had ethnic Chinese parents; (ii) aged 18 years or older; (iii) had at least 1 child aged between 3 and 17 years; (iv) could read Chinese; (v) received primary education or higher; and (vi) had a mobile phone that could receive text messages. The social workers of the participating IFSCs obtained informed written consent from all individual participants of their centers.

### Intervention

#### Pre-intervention Phase

A working committee was formed (comprising a public health academic, a medical officer, a nurse, and three registered social workers) to co-design and refine the intervention and questionnaires through a two-phase process to enhance the feasibility, relevance, and appropriateness of the intervention and questionnaires. Phase 1 included a pre-study discussion group of 10 frontline social workers who commented on the first version of intervention and questionnaires in May 2015. Based on their comments, the intervention and questionnaires were modified. Phase 2 included a pilot trial conducted for 18 community participants in June 2015 to assess the acceptability and comprehensiveness of the second version of intervention and questionnaires. The format and content of the intervention and questionnaires were finalized by the working committee after reviewing and incorporating community participants' comments and suggestions.

#### Intervention Phase

**Figure 1:**
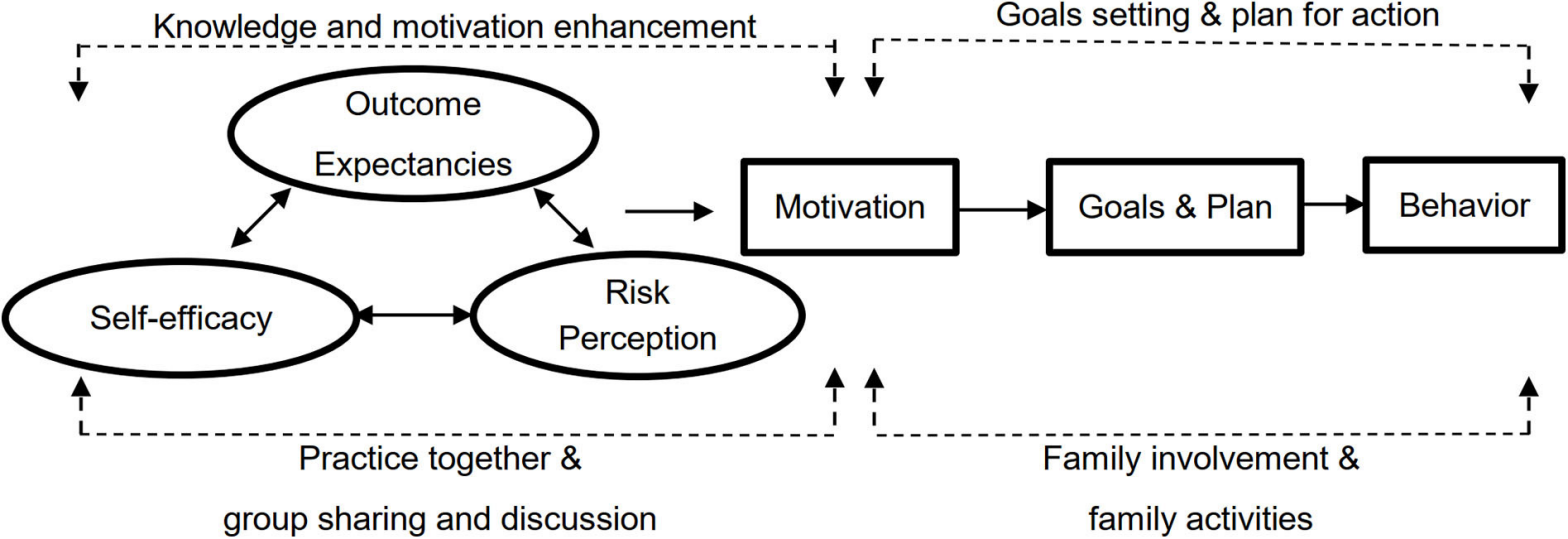
The essential components and strategies of the theory-based intervention.

Two social workers from the working committee conducted the FEP at the eight Caritas–Hong Kong IFSCs from July 2015 to September 2016. In the experimental group, 11 Zero-time exercise intervention classes were implemented for 357 participants from four IFSCs; in the control group, 12 healthy eating information classes were conducted for 316 participants from the other four IFSCs. Each class recieved three face-to-face sessions totalling 6 h and 30 min and 16 text messages as part of the intervention, and a post-intervention feedback collection session. Figure 1 shows the essential components and strategies of the theory-based intervention. Table 1 shows the objectives of each session of the experimental and control groups.

**Table 1:** The objectives of each session for the experimental group and control group.

**Sessions**	**Experimental group**	**Control group**
	**Physical activity (PA) intervention**	**Healthy eating information**
Baseline (Session I) knowledge and motivation enhancement session (2.5 h)	To increase participants' health awareness and risk perception of of physical inactivity (30 min); To introduce ZTEx and enhance participants' confidence toward exercising regularly (1 h); To help set realistic desired outcomes of regular Zero-time Exercise (30 min); and To set exercise goals and make plans for action (30 min).	To increase participants' health awareness and risk perception of diabetes and overweight (30 min); To enhance participants' confidence toward having a healthy diet (1 h); To help set realistic desired outcomes of having a healthy diet (30 min); and To set goals and make plans for implementing a healthy diet (30 min).
3-month follow-up (Session II) experience sharing session (1.5 h)	To assess the effectiveness of the intervention (15 min); To strengthen participants' intrinsic motivation for actively doing physical activity (25 min); To enhance participants' self-efficacy for doing ZTEx regularly (25 min); and To enhance participants' confidence in being role models for exercising regularly for their family members (25 min).	To assess the effectiveness of the intervention (15 min); To strengthen participants' intrinsic motivation for having a healthy diet regularly (25 min); To enhance participants' self-efficacy for having a healthy diet regularly (25 min); and To enhance participants' confidence in being role models for having a healthy diet for their family members (25 min).
6-month follow-up (Session III) family involvement session (2.5 h)	To assess the effectiveness of the intervention (15 min); To directly introduce ZTEx to family members (45 min); and To provide joyful, memorable family time and family game time (1 hour 30 min).	To assess the effectiveness of the intervention (15 min); To directly introduce healthy dietary habits to family members (45 min); and To provide joyful, memorable family time and family game time (1 hour 30 min).
1-year follow-upfeedback collection session and holistic health talk(2.5 h)^*^	To assess the effectiveness of the intervention (15 min); and To highlight the importance and methods of enhancing holistic health and introduce information on healthy eating (2 h and 15 min).	To assess the effectiveness of the intervention (15 min); and To highlight the importance and methods of enhancing holistic health and introduce information on ZTEx (2 h and 15 min).

*Zero-time exercise (ZTEx) refers to simple strength- and stamina-enhancing physical activity*.

* *Post-intervention feedback collection session was not part of the intervention*.

#### Experimental Group

In the experimental group, Session I was a 2 h and 30 min “knowledge and motivation enhancement” session (July to September 2015). We first enhanced participants' risk perception by discussing the likely consequences of physical inactivity and promoted exercise self-efficacy by introducing ZTEx. We aimed to increase participants' intrinsic motivation for being active and demonstrated how the exercises, such as raising both heels while standing, raising both feet and legs off the ground while sitting, or stretching, could be integrated into daily life. The participants were encouraged to access the ZTEx YouTube videos with different themes (e.g., for students, https://www.youtube.com/watch?v=mCTd37xEk5s; elderly, https://www.youtube.com/watch?v=EJXR0crHjZA&t=47s; integration of daily life, https://www.youtube.com/watch?v=vnKqDrHsP8k; and family games, https://www.youtube.com/watch?v=JMd_D2ndFJU), for the details of ZTEx physical activity components, movements, and applications. The participants were encouraged to share the links with their family members and do daily exercises together with family members.

We helped participants to come up with realistic expectations by sharing our personal experiences (i.e., benefits and barriers) with regular ZTEx. Participants were encouraged to share what they had learned and engage their family members through ZTEx and fun family games. We highlighted the importance of praise when involving family members through exercise because showing appreciation has been recommended as a way to strengthen family communication (33). Prior to the end of the session, we asked the participants to set their exercise goals, provide activity details (e.g., time, types of exercises, targeted family members), and document this plan on a take-home exercise record worksheet. Goal-setting has been reported to facilitate the self-regulation of physical activity behavior (34), and self-monitoring was identified as a promising strategy to increase physical activity (35). A physical activity kit was given to each participant. The kit comprised a leaflet with pictorial instructions for basic exercise movements, a 1-liter dumbbell-shaped water bottle and a handgrip to serve as reminders to do ZTEx regularly, and a magnetic clip to hold the exercise record worksheet. Participants were expected to record their daily ZTEx and exercise with family members on the record worksheets, which also served as reminders to maintain exercise habits.

Session II was conducted 3 months after the initial session (October to December 2015) and was a 1 h and 30 min “experience sharing” session. We highlighted successful examples and feelings of achievement, discussed the barriers to doing physical activity, and explored various solutions to these barriers with the participants to enhance their exercise motivation, goal-setting, and action-planning for regular physical activity. We encouraged the participants to actively participate in the session by getting involved in discussions and sharing their experiences, since an active approach has been shown to be more effective than a passive approach involving didactic educational talks (36). As a result, the participants gained confidence in becoming role models for their family members (25, 31).

Session III, which took place 6 months after the initial session (January to March 2016), was a 2 h and 30 min “family involvement” session. A maximum of three additional family members per participant were invited to join this session. We introduced ZTEx to the participants' family members and actively encouraged them to do ZTEx together, using a game-based approach, by completing tasks from the on-site game sheets. The last session was a 2 h and 30 min “feedback collection” session at 1 year after the initial session (July to September 2016). This 1-year follow-up session was not part of the intervention. It aimed to collect feedback from the participants and provide holistic health information; in the case of the experimental group, information on healthy eating was provided.

As part of the FEP, 16 text messages were sent to the participants, including six monthly text messages in the first half of the study and 10 bi-weekly text messages in the second half of the study. Supplementary Table 1 shows the text messages for the experimental and control groups. Text messages for the experimental group were focused on physical activity. We encouraged the participants to share the text messages with their families. Reinforcements created by text messaging and periodic prompts may increase the likelihood of exercising (37), and periodic prompts have been recognized as an effective method to encourage and reinforce healthy behavior (38).

#### Control Group

Content for the control group was focused on healthy eating rather than physical activity and ZTEx. The control group received the same number of sessions as the experimental group, on the same schedule, and with the same total duration. The control group also received an identical number of text messages as the experimental group. However, the text messages for the control group were focused on healthy eating.

#### Fidelity Checks

For each intervention session, two staff members (one from the academic institution and one from the IFSC) independently completed fidelity checklists for the session. The fidelity assessment aimed to standardize the quality of the intervention, including the key components to cover and the time spent on each component. The listed objectives for each session were achieved and completed within the expected period.

### Data Collection

Self-administered questionnaires were used at baseline and at the 3-month, 6-month, and 1-year assessments. One member from each participant's family also completed a brief questionnaire at the 6-month assessment. Four focus group interviews were conducted to obtain participants' feedback after the completion of the FEP in September 2016. Participants' feedback on the quality of the intervention content and on-site observations of participants' responses to the intervention were collected for triangulation of the qualitative and quantitative findings.

### Measures

#### Simple Strength- and Stamina-Enhancing Physical Activity (ZTEx), Sedentary Behavior, and Physical Activity and Fitness

Participants' engagement in simple strength and stamina-enhancing physical activity was assessed by asking two questions. The first asked the number of days on which the participant had engaged in such physical activity during the prior 7 days; responses ranged from “0 days” to “7 days.” The second question asked the time spent doing ZTEx on one of those days; responses were categorized into units of time (one unit of time was ≤15 min), including: “0 = none,” “1 = ≥1 - <15 min per day,” “2 = ≥15 - <30 min per day,” and “3 = ≥30 min or more per day.” The units of time spent doing ZTEx during the prior 7 days was calculated by multiplying the units of time of spent doing ZTEx with the number of days on which the participants had engaged in ZTEx during the prior 7 days.

Questions from the short form of the International Physical Activity Questionnaire—Chinese version (IPAQ-C) were used to assess participants' sedentary behavior and physical activity by asking for their self-reported sitting time and the number of days on which they engaged in at least 10 min of moderate and vigorous physical activity, respectively (21). The questions were: “On a typical weekday in the last 7 days, how many h per day did you typically spend seated?”; “During the last 7 days, on how many days did you do at least 10 min of moderate physical activity?”; and “During the last 7 days, on how many days did you do at least 10 min of vigorous physical activity?” The internal reliability of the Chinese version of the questionnaire was high, with an intraclass correlation coefficient of 0.79 (39).

A foot-pedaling physical fitness performance game was conducted at the beginning of each session. The participants were required to sit on a stable chair (about 43 cm in height) with their back touching the seat pan, arms, and hands held straight down by their sides, hips flexed, knees slightly bent, and hamstrings lifted off the chair. The participants were required to pedal (as if on an imaginary bicycle) with a rhythm of ~1 cycle per second. Their hamstrings should not touch the chair, and their soles should not touch the ground during the process. The participants counted and recorded the number of cycles of foot-pedaling completed in 2 min.

#### Sweetened Beverage Consumption

We assessed sweetened beverages consumption by asking the number of days on which participants consumed sweetened beverages in the last 7 days. Responses ranged from “0 days” to “7 days.”

#### Family Communication

We assessed the extent to which participants involved family members by asking two questions: “During the last 7 days, on how many days did you encourage your family to do simple strength- and stamina-enhancing physical activity?”; and “During the last 7 days, on how many days did you do simple strength- and stamina-enhancing physical activity with your family?.” The responses ranged from “0 days” to “7 days.”

#### Perceived Well-Being

Perceived well-being was assessed by asking three questions related to health, happiness, and family harmony (40): “Do you think that you are happy?”; “Do you think that you are healthy?”; and “Do you think that your family is harmonious?.” The responses to each item ranged from 0 (not happy/healthy/harmonious) to 10 (totally happy/healthy/harmonious).

#### Family Members' Simple Strength and Stamina-Enhancing Physical Activity Practice

At the 6-month follow-up, one family member (aged 18 years or older) from the participant's family reported the number of days on which they had done simple strength- and stamina-enhancing physical activity by themselves in the last 7 days. The responses ranged from “0 days” to “7 days.”

#### Reactions to the Intervention Content and Design

We asked participants to grade the quality and utility of the intervention and its contents. The participants were asked “How much did you like the intervention?” and “How feasible is it to incorporate the exercises you have learned into your daily life?” Responses were made on an 11-point Likert scale, ranging from 0 (very unsatisfied/totally not feasible) to 10 (very satisfied/very feasible). Participants were also asked “Would you recommend this workshop to your friends and family?” with response options of “Yes” or “No.”

### Statistical Analysis

The calculation of the sample size was conducted by comparing the number of days in which the experimental group and control group did simple strength- and stamina-enhancing physical activity in the 7 days prior to filling out the questionnaire at the 3-month assessment. To detect a medium effect size of 0.5 with 80% power and a 5% false-positive rate, we needed 80 individuals per group. We took the intracluster correlation as 0.05 to account for the clustering effect of the IFSCs. With eight IFSCs, we needed 84 participants per group under each IFSC. Allowing for 10% attrition, we needed 352 individuals per group.

Analyses were conducted using Stata (version 13.0). All significance tests were two-sided with *p* < 0.05 indicating statistical significance. An intention-to-treat (ITT) analysis was conducted, with missing values replaced by the baseline values of the outcome variables. A mixed-effects model was used to examine the intervention effects of the PA intervention. With this mixed-effects model method, (i) the extra covariance between repeated measurements was taken at the baseline, 3-month, 6-month and 1-year assessments; (ii) the clustering effect of individuals under the same IFSC and class was treated as a random effect; and (iii) the baseline values of the outcome variables were treated as covariates. We first examined the consistency of the intervention effect over time by testing for the significance of the interaction term of group-by-time in the analysis. A significant interaction effect meant that there were significant differences between groups over time. Where evidence of a group-by-time interaction effect was found, the intervention effects at the 3-month, 6-month, and 1-year assessment are reported separately. Where no interaction effect was found, the overall intervention effect is reported.

Participants' demographic characteristics, including marital status, educational level, and monthly household income significantly differed between the experimental group and control group; these were considered to be potential confounders (Table 2). Sensitivity analyses were conducted, including (i) an ITT analysis with adjustments for the potential confounders (e.g., age, sex, marital status, educational level, monthly household income); (ii) a complete case analysis on those who completed all assessments at baseline, and the 3-month, 6-month, and 1-year follow-ups; and (iii) a complete case analysis that adjusted for the potential confounders.

**Table 2:** Characteristics of all participants, participants who completed 1-year follow-up, participants who participated in the focus group interviews, and participants who did not participate in the focus group interviews (*n* = 673).

	**All participants**			**Participants who completed the 1-year follow-up**			**Focus group interviews**		
	**Experimental group (*n* = 357)**	**Control group (*n* = 316)**	***p*-value**	**Experimental group (*n* = 309)**	**Control group (*n* = 284)**	***p*-value**	**Participated (*n* = 32)**	**Did not participate (n= 641)**	***p*-value**
	***n*** **(%)**	***n*** **(%)**		***n*** **(%)**	***n*** **(%)**		***n*** **(%)**	***n*** **(%)**	
Sex									
Female	327 (92)	293 (93)	0.59	265 (92)	264 (93)	0.52	31(97)	589 (92)	0.31
Male	30 (8)	23 (7)		44 (8)	20 (7)				
Age			0.62						
18–<30 years	12 (3)	13 (5)		8 (2)	9 (3)	0.85	0 (0)	25 (4)	0.52
30–39 years	160 (45)	149 (47)		139 (45)	134 (47)		15 (47)	294 (46)	
40–49 years	150 (42)	118 (37)		129 (42)	109 (38)		15 (47)	253 (39)	
≥50 years	35 (10)	36 (11)		33 (11)	32 (11)		2 (6)	69 (11)	
Education level			<0.001^***^						
Primary and below	33 (9)	64 (20)		31 (10)	61 (22)	<0.001^***^	3 (9)	94 (15)	0.41
Secondary and tertiary	270 (91)	252 (80)		278 (90)	223 (78)		29 (91)	547 (85)	
Marital status			<0.001^***^						
Married	275 (77)	203 (64)		240 (78)	180 (63)	<0.001^***^	25 (78)	452 (71)	0.36
Widowed/divorced/unmarried	82 (23)	113 (36)		69 (22)	104 (37)		7 (22)	189 (39)	
Household monthly income			<0.001^***^						
CSSA and < HK$10,000	119 (34)	161 (53)		103 (33)	147(52)	<0.001^***^	10 (31)	270 (42)	0.22
HK$10,000 or more	238 (66)	155 (47)		206 (67)	137 (48)		22 (69)	371 (58)	

Between group comparisons:

*** *p < 0.001*.

*CSSA, Comprehensive Social Security Assistance*.

*US$1 = HK$7.8*.

The focus group interviews were conducted by an experienced researcher from the working committee. All qualitative interviews were audiotaped and transcribed verbatim in Chinese. Two project members, one of whom had attended the interviews, coded the transcripts. The transcripts were analyzed using thematic framework analysis, following the guidelines recommended by Morse and Field (41). A mixed-methods design was used to interrelate and interpret the qualitative and quantitative data to validate the results (42).

## Results

### Participants

**Figure 2:**
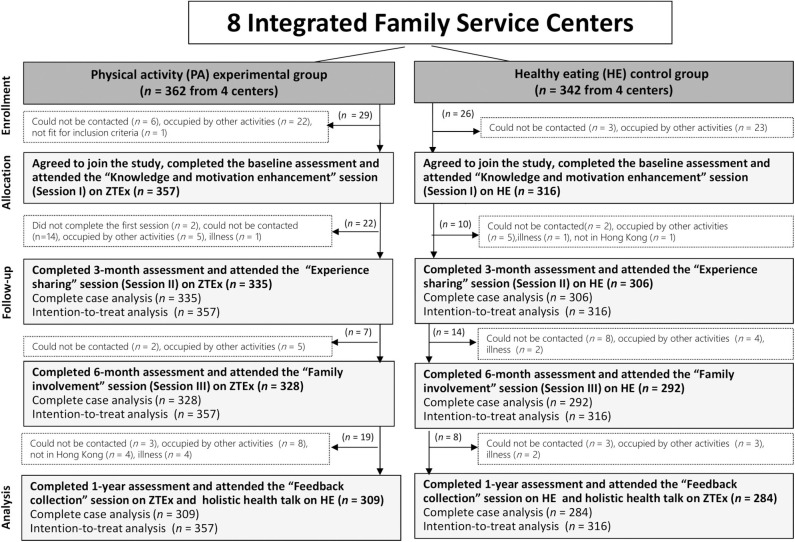
Flow of participants.

Of the 728 participants who registered for the FEP, 673 participants (92% female and 46% aged 30–39 years) attended Session I as part of the experimental group (*n* = 357) and control group (*n* = 316) and were included in the analysis. Thirty-two participants (22 from the experimental group and 10 from the control groups) were absent from Session II, and 21 participants (7 from the experimental group and 14 from the control group) were absent from Session III. Twenty-seven participants (19 from the experimental group and 8 from the control group) did not attend the post-intervention meeting at the 1-year follow-up. The remaining 593 participants completed the assessments at all time points. Figure 2 shows the flow of the participants. Table 2 shows significant differences in educational level, marital status, and monthly household income between the experimental and control groups. No significant differences in participant characteristics were observed between those who participated in the focus group interviews and those who did not. No harm or unintended effects were detected in either group.

### Changes in Simple Strength- and Stamina-Enhancing Physical Activity (ZTEx), Sedentary Behavior, Physical Activity, and Fitness

**Figure 3:**
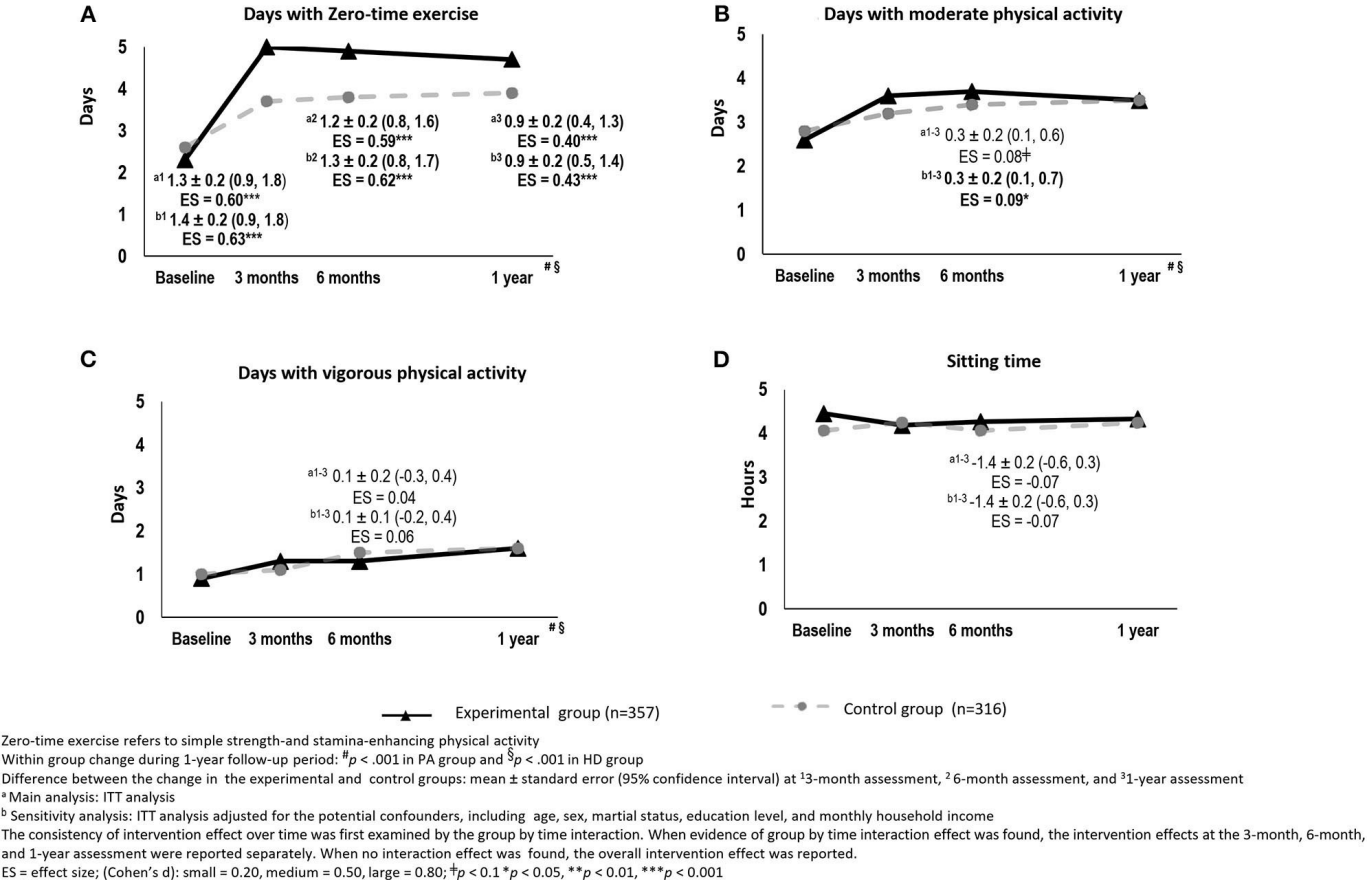
The changes in physical activity between the experimental and control groups over time [**(A)** Days with Zero-time exercise, **(B)** Days with moderate physical activity, **(C)** Days with vigorous physical activity, and **(D)** Sitting time]: intention-to-treat analysis.

Both groups reported significant increases in ZTEx and physical activity (*p* < 0.001) but no significant changes in time spent sitting (*p* > 0.05) at all time points. Compared with the control group, the experimental group reported significantly greater increases in days engaged in ZTEx: 1.3 days at 3 months (95% confidence interval [CI]: 0.9, 1.8, *p* < 0.001), 1.2 days at 6 months (95% CI: 0.8, 1.6, *p* < 0.001) and 0.9 days at 1 year (95% CI: 0.4, 1.3, *p* < 0.001). The effect sizes ranged from small to medium (Cohen's *d*: 0.40–0.60) (Figure 3A). Compared with the control group, the experimental group reported significantly greater increases in time engaged in ZTEx: 4.3 units of time (one unit of time is <15 min increase) at 3 months (95% CI: 3.1, 5.5, *p* < 0.001), 2.6 units of time at 6 months (95% CI: 1.4, 3.7, *p* < 0.001), and 1.6 units of time at the 1 year with small to moderate effect sizes than the control group (95% CI: 0.9, 3.3, *p* < 0.001, Cohen's *d*: 0.37–0.76) (Supplementary Figure 1).

However, compared with the control group, the experimental group only reported a marginally significantly greater increase, of 0.3 days spent doing moderate physical activity, with small effect size (Cohen's *d*: 0.08), at all time points (95% CI: 0.1, 0.6, *p* = 0.079) (Figure 3B). There were no significant differences in the changes in vigorous physical activity and sitting time between the two groups (Figures 3C,D).

In terms of physical fitness, compared with the control group, the experimental group showed a significantly greater increase of 20 s in the duration of foot pedaling with large effect size at the 1-year assessment (95% CI: 7.9, 31.3, *p* < 0.001, Cohen's d: 1.73), but not at the 3 and 6-month assessments (Supplementary Figure 2).

At the 1-year focus-group interviews, participants in the experimental group reported that laziness was a significant barrier to maintaining their exercise habits. They reported having a more active lifestyle than before receiving the intervention.

“[Zero-time] exercise is excellent and could be widely promoted. However, my laziness made it difficult for me to establish my exercise habit.” (A housewife, 40–49 years old).

“Before I knew about [Zero-time] exercise, I was not aware that we could perform the physical activity while waiting for the bus. Now I know I can exercise, particularly during my waiting time.” (A female clerk, 40–49 years old).

### Change in Sweetened Beverage Consumption

Both groups reported a significant decrease in sweetened beverage consumption (*p* < 0.05). The control group showed a significantly greater reduction by 0.4 days on which sweetened beverages were consumed at all time points with small effect size (95% CI: 0.01, 0.9, *p* < 0.05, Cohen's *d*: 0.28), compared with the experimental group.

### Changes in Family Communication

**Figure 4:**
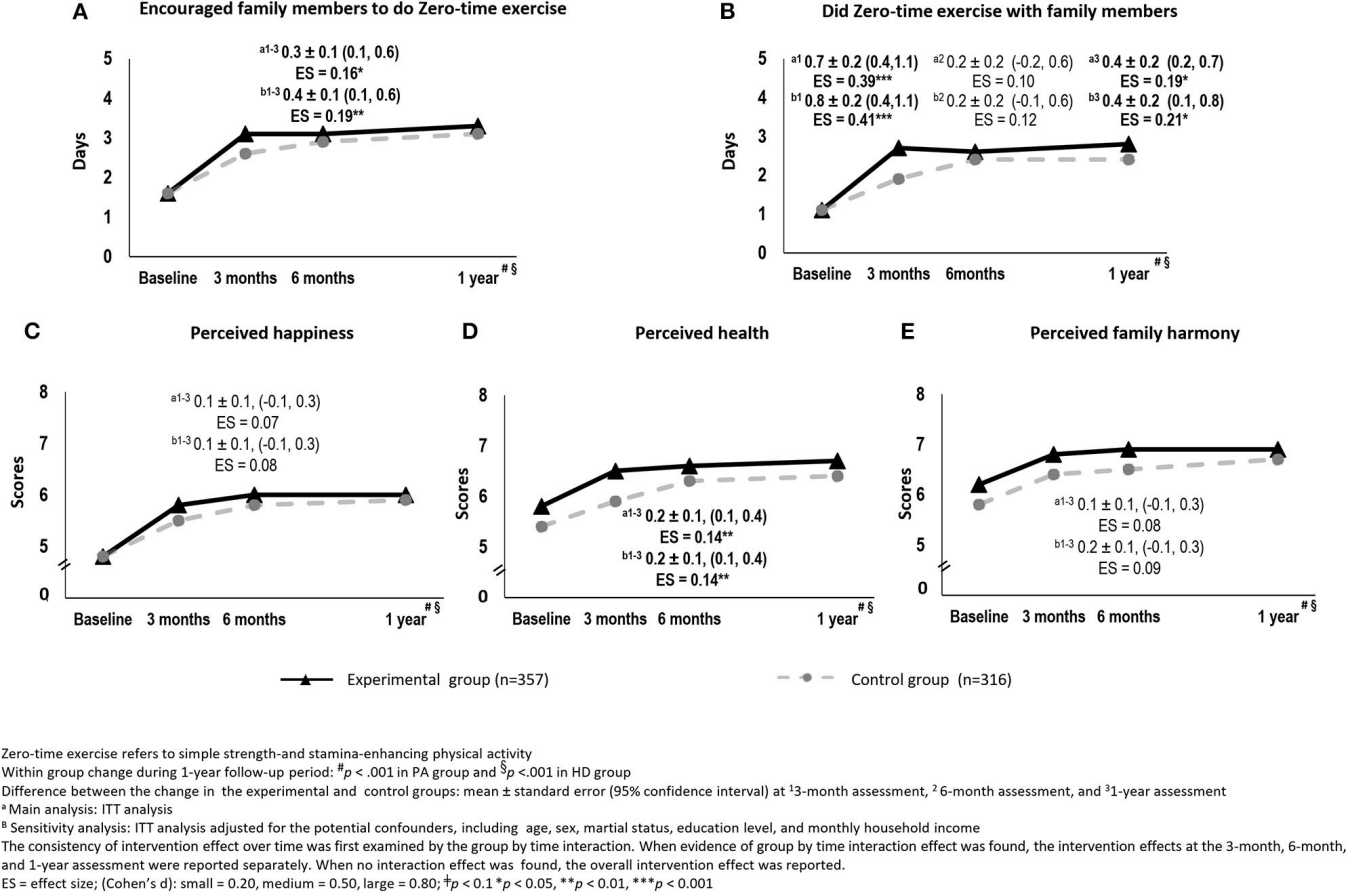
The changes in family communication and perceived well-being between the experimental and control groups over time [**(A)** Encouraged family members to do Zero-time exercise, **(B)** Did Zero-time exercise with family members, **(C)** Perceived happiness, **(D)** Perceived health, and **(E)** Perceived family harmony]: intention-to-treat analysis.

Both groups reported significant increases in the number of days spent encouraging family members to do ZTEx and doing ZTEx with their family members at all time points (*p* < 0.001). The experimental group reported a significantly greater increase of 0.3 days spent encouraging family members to do ZTEx, with a small effect size (Cohen's *d*: 0.16), than the control group at the 3-month assessment (95% CI: 0.1, 0.6, *p* < 0.05). The intervention effect was sustained at the 6-month and 1-year assessments (Figure 4A). The experimental group reported significantly greater increases of 0.7 and 0.4 days spent doing ZTEx with their family members, with small effect size (Cohen's *d:* 0.19–0.39), at the 3-month and 1-year assessments (95% CI: 0.4, 1.1, *p* < 0.001 and 95% CI: 0.2,0.7, *p* < 0.05, respectively) (Figure 4B).

At the 1-year focus-group interviews, participants stated that ZTEx was an interesting topic to discuss with their family members. The participants considered themselves good role models for their family members in terms of integrating simple strength- and stamina-enhancing physical activity into their daily lives.

“I told my son that ZTEx could improve his flat feet and reduce his back pain; he showed great interest in it.” (A housewife, 40–49 years old).

“Our relationship was better. At least, we exercised together and had more topics for discussion.” (A housewife, 40–49 years old).

### Changes in Perceived Well-Being

Both groups reported significant improvements in perceived happiness, health, and family harmony (*p* < 0.001) at all time points. The experimental group showed significantly greater improvement than the control group of 0.2 in perceived health, with a small effect size (Cohen's *d*: 0.14) at the 3-month assessment (95% CI: 0.1, 0.4, *p* < 0.05). The intervention effect was sustained at the 6-month and 1-year assessments (Figure 4D). However, there were no significant differences in the improvement in perceived happiness and family harmony between the two groups (Figures 4C,E).

At the 1-year focus-group interviews, the participants reported improved health, fitness, happiness and emotional control because of regular physical activity.

“After having regular exercise, I felt happier and more energetic than before. My health was improved and blood pressure was better.” (A housewife, 30–39 years old).

“[I] walked more than before joining the program and I am much healthier than before.” (A female part-time worker, 30–39 years old).

The improvements in perceived well-being (including perceived health, happiness, and family harmony) showed significant positive associations with (i) the increases in days spent doing physical activity (including ZTEx, moderate physical activity and vigorous physical activity) and (ii) the increases in days spent encouraging family members to do ZTEx and doing ZTEx with family members (Table 3).

**Table 3:** The associations between participants' changes in physical activity and family communication and the changes' in well-being at different time points (n = 673).

***n*** **=** **673**		**Changes in physical activity^#^**				**Changes in family communication^#^**	
		**ZTEx**	**Moderate physical activity**	**Vigorous physical activity**	**Sitting time**	**Encouraged family members to do ZTEx**	**Did ZTEx with family members**
**Changes in perceived health^#^**							
At 3-month	*r*	0.179^***^	0.149^***^	0.125^***^	−0.029	0.151^***^	0.154^***^
	*p*	<0.001	<0.001	0.001	0.451	<0.001	<0.001
At 6-month	*r*	0.092^*^	0.082^*^	0.061	0.018	0.138^***^	0.097^*^
	*p*	0.017	0.033	0.115	0.635	<0.001	0.011
At 1-year	*r*	0.110^**^	0.150^***^	0.147^***^	−0.012	0.156^***^	0.150^***^
	*p*	0.004	<0.001	<0.001	0.765	<0.001	<0.001
**Changes in perceived happiness^#^**							
At 3-month	*r*	0.145^***^	0.049	0.085^*^	−0.043	0.152^***^	0.124^***^
	*p*	<0.001	0.201	0.028	0.271	<0.001	0.001
At 6-month	*r*	0.083^*^	0.079^*^	0.145^***^	−0.064	0.129^**^	0.087^*^
	*p*	0.031	0.042	<0.001	0.098	0.001	0.024
At 1-year	*r*	0.157^***^	0.139^***^	0.217^***^	−0.008	0.166^***^	0.190^***^
	*p*	<0.001	<0.001	<0.001	0.842	<0.001	<0.001
**Changes in perceived family harmony^#^**							
At 3-month	*r*	0.099^*^	0.034	0.067	−0.016	0.147^***^	0.108^**^
	*p*	0.010	0.385	0.082	0.680	<0.001	0.005
At 6-month	*r*	0.065	0.028	0.118^**^	0.006	0.165^***^	0.105^**^
	*p*	0.093	0.469	0.002	0.878	<0.001	0.007
At 1-year	*r*	0.129^**^	0.084^*^	0.157^***^	0.036	0.174^***^	0.159^***^
	*p*	0.001	0.029	<0.001	0.346	<0.001	<0.001

# *The change from baseline to the specific time point*.

*ZTEx, Zero-time exercise refers to simple strength-and stamina-enhancing physical activity*.

*The association between two variables was compared by Pearson correlation*.

*r = correlation coefficient*;

* *p < 0.05*,

** *p < 0.01*,

*** *p < 0.00*.

### Family Members' Practice of Simple Strength- and Stamina-Enhancing Activity

In the experimental group, 253 and 166 participants returned the take-home exercise record worksheets at the 3- and 6-month follow-ups, respectively. The homework returned by participants also showed that they and their children did ZTEx at home, indicating acceptance.

At the 6-month follow-up, a total of 620 families (2,480 participants and their family members) joined the family involvement session; 346 family members (one family member per participant, aged 18 years or older) answered the brief questionnaire for family members. The demographics of family representatives and their relationships with principal participants did not differ significantly between the groups (experimental group: *n* = 256, 57% male, 58% aged ≥30–50 years, 52% were spouse; control group: *n* = 90, 49% male, 54% aged ≥30– <50 years, and 49% were spouse). Family members in the experimental group did significantly more simple strength- and stamina-enhancing activity than those in the control group, with a small effect size (mean ± SD: 2.9 ± 2.4 days vs. 2.2 ± 2.4 days, *p* < 0.05; Cohen's *d*: 0.27) (Table 4). From our unobtrusive observation, participants and their children were actively engaged, enthusiastically followed the ZTEx demonstration, and showed enjoyment.

**Table 4:** Demographic characteristics of family representatives who answered the brief family questionnaire and their relationship with principal participants (*n* = 346).

	**Experimental group**	**Control group**	***p*-value**
	***n* = 256**	***n* = 90**	
	***n* (%)**	***n* (%)**	
**Sex**			
Male	151 (57.0)	45 (48.9)	
Female	114 (43.0)	47 (51.1)	0.18
**Age group**			
<30 years	40 (15.6)	20 (22.0)	
≥30– <50 years	149 (58.0)	49 (53.8)	
≥50 years	68 (26.5)	22 (24.2)	0.38
**Relationship with principal registered participants**			
Spouse	133 (52.4)	44 (48.9)	
Parents or parents-in-law	33 (13.0)	11 (12.2)	
Sons or daughters	54 (21.3)	26 (12.2)	
Sisters or brothers	4 (1.6)	1 (1.1)	
Friends or other relatives	30 (11.8)	8 (8.9)	0.63
	Mean ± SD	Mean ± SD	
**Days engaged in ZTEx**	2.9 ± 2.4	2.2 ± 2.4	0.024^*^

Between group comparisons:

* *P < 0.05*.

### Reactions to Intervention Content and Design

At the 1-year assessment, participants rated both the quality and utility of the intervention content a score of 9.0 ± 1.2. All participants reported that they would recommend this intervention programme to their friends and families.

The participants reported that the PA intervention content was comprehensive and practical. Remedial classes offered flexibility to those who were unable to attend the scheduled sessions. The text messages reminded the participants to do regular exercise by themselves and with their family members.

“The [ZTEx] content was simple and easy to understand, and the examples of ZTEx (such as standing with raised heels) were convenient to apply in my daily routine” (A housewife, 40–49 years old).

“When I saw the calendar worksheet, I remembered to do [ZTEx], then [I would] practice a while.” (A female employee, 40–49 years old).

“Electronic messages always reminded us to do [ZTEx].” (A housewife, 30–39 years old).

After performing the main analysis (i.e., the ITT analysis without adjusting for potential confounders), we conducted sensitivity analyses to assess the consistency of the findings. The ITT analysis with adjustment for potential confounders yielded similar findings to the main analysis, except for the intervention effect on moderate physical activity. The experimental group reported significantly greater improvements in this regard (by 0.3 days), with a small effect size (Cohen's *d*: 0.09) at the 3-month assessment (95% CI: 0.1, 0.7, *p* < 0.05). The intervention effect was sustained at the 6-month and 1-year assessments (Figures 3, 4).

The complete case analyses, with and without adjustment for potential confounders, also showed similar findings to those of the main analysis, except for the findings in relation to moderate physical activity. The experimental group reported significantly greater improvements in this regard (by 0.5–0.6 days), with small effect sizes (Cohen's *d*: 0.24–0.26) at the 3-month assessment (95% CI: 0.1, 1.0, *p* < 0.05). The intervention effect was sustained at the 6-month and 1-year assessments (Supplementary Figures 1–3). Compared with the control group, the experimental group reported significantly greater improvements by scores of 0.2 in personal happiness (95% CI: 0.1, 0.4, *p* < 0.05; Cohen's *d*: 0.14) and 0.2 in family harmony (95% CI: 0.1, 0.4, *p* = 0.037; Cohen's *d*: 0.16), with small effect sizes, at the 3-month assessment. The intervention effect was sustained at the 6-month and 1-year assessments (Supplementary Figure 4).

## Discussion

This cRCT demonstrated that the PA intervention was effective in enhancing physical activity, family communication, and perceived health among deprived families in Hong Kong. This intervention showed the benefits of simple stamina- and strength-enhancing physical activity, the feasibility of using low-cost methods to have regular exercise, and the applicability of conducting a community-based physical activity intervention. The qualitative data provided additional evidence to support the effectiveness of this intervention.

The intervention used to enhance physical activity differs significantly from most of the interventions reported in the extant literature. The interventions in the literature comprised 18 sessions (16), 16 sessions (9), 8 sessions (10, 14, 43), and 5 sessions (11, 13). Our intervention comprised three face-to-face sessions (totalling 6 h and 30 min) and 16 text messages, making it shorter than most of the interventions in the extant literature. With the advancement of information communication technology and high levels of mobile phone usage in Hong Kong, we made good use of text messaging to promote physical activity. Text messages have been recognized as effective reminders and an important method to deliver health-related information to individuals because it reduces the barriers of situational constraints (44, 45) and offers a cost-effective and acceptable method to deliver health education and promotion (46).

Our intervention used a foot-in-the-door approach, a compliance tactic to start with the easiest first step, the idea being that small demands are easier to meet (47). This approach has been applied in various fields such as the promotion of tobacco control and regular physical activity (48, 49). We promoted integrating simple strength- and stamina-enhancing physical activity into daily life and advocated that performing some physical activity (even a light amount) is better than not performing any physical activity. This belief is consistent with the recommendations of the 2018 Physical Activity Guidelines Advisory Committee Scientific Report (23).

We also emphasized that ZTEx could be easily personalized with no extra cost. This is important as barriers of money and time have been reported as critical deterrents when initiating exercise (50), particularly in deprived groups. Our PA intervention requires few resources to disseminate and is easily applicable to various settings, particularly in cities with limited space, such as Hong Kong. Our intervention was well-accepted by the parents in Hong Kong, although the majority of parents and children tend to focus on academia rather than exercise and are often preoccupied with daily tasks (18). The acceptance of the intervention may be attributed to the feasibility of the suggested exercises, which can be done at home and in office settings and thus easily integrated into daily lives. The well-structured curriculum of this intervention is easy to replicate and implement for further research. The current study also showed positive associations between increased ZTEx engagement by oneself and with one's family as well as improvements in well-being in terms of perceived health, happiness, and family harmony at all time points. These findings suggest that this community-based intervention may have potentially significant positive effects on mental and physical outcomes.

We acknowledge that there are certain limitations to the study. First, since the majority of our participants were females (only 8% of FEP participants were male), the findings would be more applicable in females than males. Second, the control group showed increases in physical activity, family interaction, and well-being. This could be due to the dissemination of a similar type of health-related information (healthy eating) in the control group, which may influence participants' health awareness. Third, considering that validated questionnaires were unavailable, we self-developed our outcome-based questions to assess the participants' practices in relation to doing simple strength- and stamina-enhancing physical activity by themselves and with their family members. Fourth, owing to resource constraints, we were unable to objectively assess the accumulated duration of physical activity; we only measured the self-reported days engaged in physical activity. Self-reported moderate and vigorous physical activity values can be higher than objective values, particularly in inactive participants (51). Fifth, as the intervention was a community-based intervention and the questionnaires had to be kept at a reasonable and manageable length for participants, we were unable to assess changes in all of the cognitive factors for the formation of exercise motivation and regulatory factors for regular physical activity. Sixth, we did not use physical activity level as inclusion criteria. Our participants could have included both people who were active and inactive, and we might have overlooked the need for more exercise for inactive participants. Seventh, fewer family members than we had participants completed the family physical activity questionnaires because the staff of some IFSCs were not aware that they needed to deliver the questionnaires at the 6-month family involvement session. Lastly, we only collected feedback from family members aged 18 years and over who joined the family sessions and did not collect feedback from all family members at all time points because of resource constraints. We did not collect feedback from family members on their changes in family happiness, health and harmony, and did not identify the additional effects of text messaging on traditional face-to-face interventions. To further understand how intervention effects can be sustained and maintained for longer periods, future studies should aim to identify specific intervention components effective for community-based intervention delivery; identify and assess changes in cognitive and regulatory factors such as risk perception and self-monitoring; and assess the frequency and interactivity of messaging, and time of delivery.

The community-based lifestyle-integrated PA intervention, using behavioral change strategies such as the foot-in-the-door approach and involving family members, was assessed through comprehensive quantitative and qualitative evaluations. The preliminary evidence showed the positive effects of the intervention on enhancing physical activity, perceived health, and family communication, and the intervention could serve as a new model to promote a healthy lifestyle in the community. The community-based lifestyle-integrated PA intervention involving family members has the potential to benefit more people and other service sectors such as elderly service.

## Data Availability Statement

The datasets presented in this article are not readily available because the sharing of data to third parties was not mentioned in subjects' consent. Requests to access the datasets should be directly contact corresponding author.

## Ethics Statement

This study involving human participants were reviewed and approved by The Institutional Review Board of The University of Hong Kong/Hospital Authority Hong Kong West Cluster. The patients/participants provided their written informed consent to participate in this study.

## Author Contributions

TL and SS led the conception and design of the intervention and assessments and were closely involved in data interpretation and manuscript revision. AL was a major contributor for the intervention design and coordination, analyzed and interpreted the data, and was responsible for drafting the manuscript. CF was a major contributor to the intervention design. AW was a major contributor to the intervention design and coordination between HKU-SPH and Caritas–Hong Kong. LH was involved in the statistical analysis of the data. EL was involved in the intervention design, oversaw the IFSC units involved in the study, and played a key role in the implementation of the intervention. DL was involved in the intervention design and played a key role in the coordination of the IFSC units involved in the study. JT coordinated the IFSC units involved in the study and played a key role in recruitment, intervention implementation, and data collection. All authors read and approved the final manuscript.

## Conflict of Interest

The authors declare that the research was conducted in the absence of any commercial or financial relationships that could be construed as a potential conflict of interest.
