# Supplementary material for: A Community-Based Lifestyle-Integrated Physical Activity Intervention to Enhance Physical Activity, Positive Family Communication, and Perceived Health in Deprived Families: A Cluster Randomized Controlled Trial
Source: Front Public Health. 2020 Sep 15;8:434. doi: 10.3389/fpubh.2020.00434 (PMC7522171; doi:10.3389/fpubh.2020.00434)
Supplement: Supplementary file 1 [file Data_Sheet_1.PDF]

Supplementray Table S1 The mobile text messages for the experiemental group and control group

Message No.	**Experiemental group** (received physical activity intervention)	**Control group** (received healthy eating information)
**M1** 1st month	**"Family Education Project" Tips:** You can perform some stretching exercises when you are sitting for a long time. Foot pedalling is a fun family game. Please add our number into your contact list.	**"Family Education Project" Tips:** Be good to yourself - read food labels before you buy, eat less sugar and more vegetables. Please add our phone number into your contact list.
**M2** 2nd month	**"Family Education Project" Tips:** Did you start your enjoyable, easy and effective ZTEx? If not, please start with your family members today. You and your family will feel much healthier.	**"Family Education Project" Tips:** Did you start your less sugar and more vegetables and fruits diet? If not, please start with your family members today. Your family will feel so much healthier.
**M3** 3rd month	**"Family Education Project" Tips:** Do you remember your exercise goals? I believe you can make it. Please bring your ZTEx record worksheet to the next session [date and time]; we have prepared a souvenir for you.	**"Family Education Project" Tips:** Do you remember your healthy diet goals? I believe you can make it. Please bring your record worksheet to the next session [date and time]; we have prepared a souvenir for you.
**M4** 4th month	**"Family Education Project" Tips:** Stretching exercises can help relieve back pain. Exercise can strengthen the muscles around your joint and improve your balance. Please forward our ZTEx video to your friends and family.	**"Family Education Project" Tips:** Please read the food labels before you buy, reduce your intake of sugary drinks, eat more fresh vegetables. Please forward the healthy diet video to your friends and family.
**M5** 5th month	**"Family Education Project" Tips:** Have you achieved your planned exercise goals? Rotating your shoulder and stretching your back can help relieve back and back muscle ache. Please do ZTEx with your family immediately.	**"Family Education Project" Tips:** Have you achieved your planned dietary goals? Eating less sugar can reduce the chance of having dental caries. Intaking more vegetables can improve your bowel movement and prevent chronic disease. Please start a healthy diet with your family immediately.
**M6** 6th month	**"Family Education Project" Tips:** You can do ZTEx when you are sitting, standing and walking. Please do it consistently and remember our meeting in the coming week [date and time].	**"Family Education Project" Tips:** Please remember to select dishes with more vegetables, have less sugary drinks when dining out, and do it consistently. Please remember our meeting in the coming week [date and time].
**M7** 7th month	**"Family Education Project" Tips:** We will send health-related information to you every two weeks. We hope you share the messages with family and friends to establish healthy living habits.	**"Family Education Project" Tips:** We will send health-related information to you every two weeks. We hope you to share the messages with family and friends to establish healthy living habits.
**M8**	**"Family Education Project" Tips:** We want you to establish healthy living habits with your family. Doing more ZTEx helps to promote health, happiness, and family harmony. Exercising with family has been recognised as the most valuable family activity to strengthen family bonding.	**"Family Education Project" Tips:** We want you to establish healthy living habits with your family. Having a healthy diet with family helps promote health, happiness, and family harmony. Cooking and dining with family is a good family time!

**M9**<br>8th month	**"Family Education Project" Tips:** We hope you and your family have a relaxing and enjoyable Easter holiday. Please make good use of this holiday to do more physical activity with your family.	**"Family Education Project" Tips:** We hope you and your family have a relaxing and enjoyable Easter holiday. Please make good use of this holiday to prepare more healthy dishes for your family.
**M10**	**"Family Education Project" Tips:** Health is important for everyone, please pay more attention to it. ZTEx helps reduce the negative impact of sitting and promotes better mood. We are collecting photos of families doing ZTEx. Please share yours with us!	**"Family Education Project" Tips:** Paying more attention to your health is important. A healthy diet helps improve body weight and health. We are collecting photos of families engaging in healthy diet and cooking. Please share the happy moments of family activities and send photos to us!
**M11**<br>9th month	**"Family Education Project" Tips:** ZTEx can be done anytime, anywhere and by anybody (3A). It is an enjoyable, easy, and effective (3E) exercise. Please do it with your families and you will be much healthier.	**"Family Education Project" Tips:** Summer time is coming. Fruits and vegetables are a good choice for health. Please enjoy your family dining time, you will be much healthier.
**M12**	**"Family Education Project" Tips:** We will meet you again after 3 months. I hope that everyone can maintain healthy living habits. ZTEx helps burn more energy, enhance cardiovascular functions, and relieve stress. Please integrate ZTEx into your daily life!	**"Family Education Project" Tips:** We will meet you again after 3 months. I hope that everyone can maintain healthy living habits. Please remember the tips of "3M", more fruits, more vegetables, more fibre; and "3L", less sugar, less salt, less fat.
**M13**<br>10th month	**"Family Education Project" Tips:** ZTEx does not require extra time, money or equipment. Do you have any plans for doing exercise with your family on the coming 3-day holiday? Please spend more time with your family to strengthen your family bonding.	**"Family Education Project" Tips:** During the hot summer, please consider having more fresh vegetables instead of soft drinks and other sugary drinks. Please spend more time with your family to strengthen your family bonding.
**M14**<br>11th month	**"Family Education Project" Tips:** Summer vacation is a good parent-child time. ZTEx can help to strengthen muscles and promote your mental health. I hope you will be fitter and healthier when we meet in the upcoming session.	**"Family Education Project" Tips:** During the summer vacation, parents and children will have more parent-child time. Please make more healthy dishes. I hope you will be fitter and healthier when we meet in the upcoming session.
**M15**	**"Family Education Project":** We will have the last session with you soon. We will prepare some goodies about holistic health for you! Please attend the last meeting [date and time].	**"Family Education Project":** We will have the last session with you soon. We will share information on holistic health with you! Please attend the last meeting [date and time].
**M16**<br>12th month	**"Family Education Project":** We hope everyone has a good start in the new school year. Please attend the last meeting [date and time].	**"Family Education Project":** We hope everyone has a good start in the new school year. Please attend the last meeting [date and time].
