# Supplementary material for: A Community-Based Lifestyle-Integrated Physical Activity Intervention to Enhance Physical Activity, Positive Family Communication, and Perceived Health in Deprived Families: A Cluster Randomized Controlled Trial
Source: Front Public Health. 2020 Sep 15;8:434. doi: 10.3389/fpubh.2020.00434 (PMC7522171; doi:10.3389/fpubh.2020.00434)
Supplement: Supplementary file 2 [file Data_Sheet_2.pdf]

Supplementary Fig. S1 The changes in time engaged in simple strength- and stamina-enhancing physical activity between the experimental and control groups over time: (a) Intention-to-treat analysis (n=673) and (b) Complete case analysis (n=593)

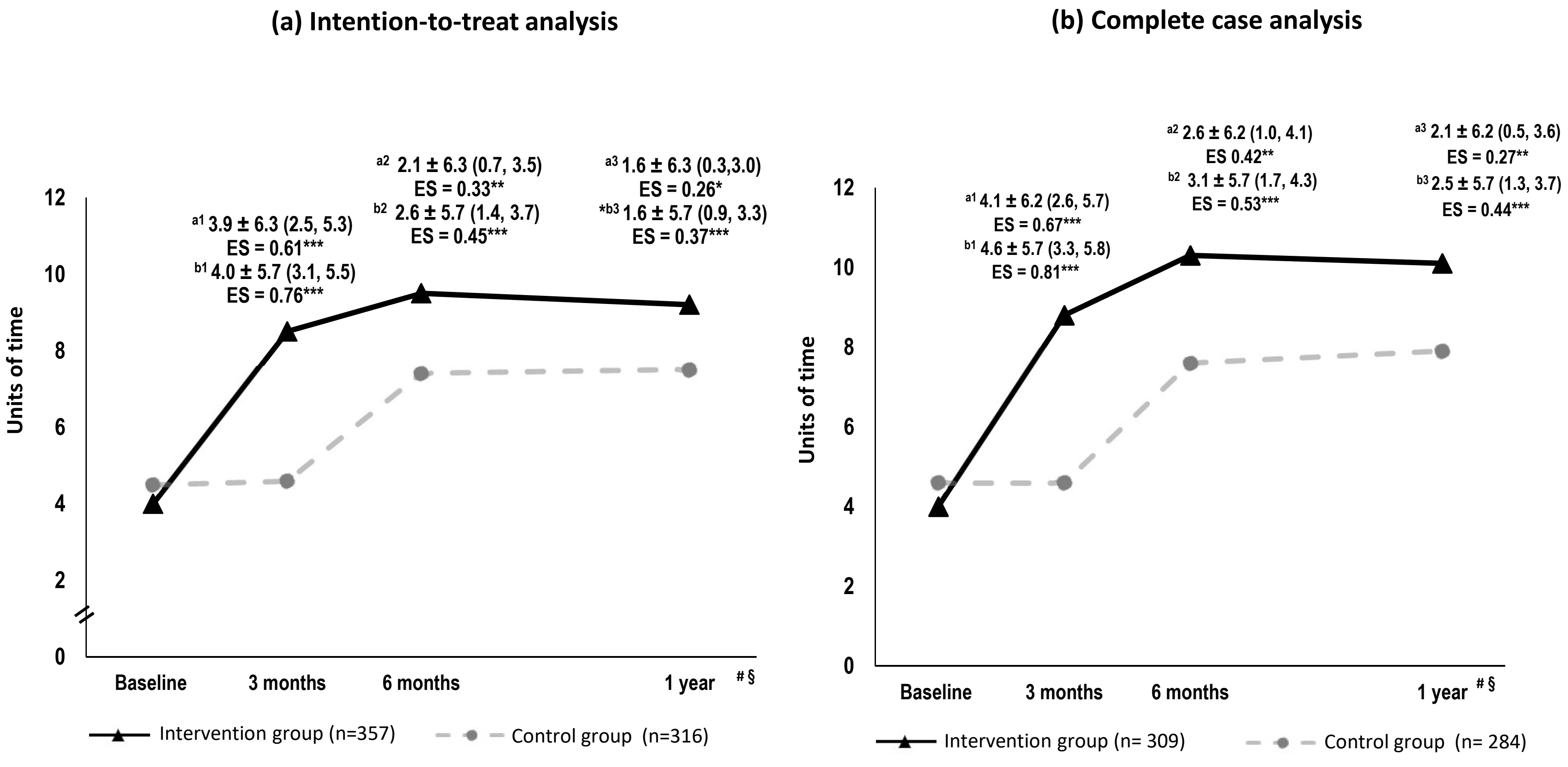

One unit of time is less than or equal to 15 minutes increase

Within group change during 1-year follow-up period: #p < 0.001 in the experimental group and §p < 0.001 in the control group

Difference between the change in the experimental group and control group: mean ± standard error (95% confidence interval) at 13-month assessment, 2 6-month assessment, and 31-year assessment

a Sensitivity analysis 2: Complete case analysis

b Sensitivity analysis 3: Complete case analysis adjusted for the potential confounders, including age, sex, martial status, education level, and monthly household income

The consistency of intervention effect over time was first examined by the group by time interaction. When evidence of group by time interaction effect was found, the intervention effects at the 3-month, 6-month, and 1-year assessment were reported separately. When no interaction effect was found, the overall intervention effect was reported.

ES = effect size; (Cohen's d): small = 0.20, medium = 0.50, large = 0.80; ǂ*p* < 0.1 **p* < 0.05, ***p* < 0.01, ****p* < 0.001

Supplementary Fig. S2 The changes in physical fitness performance (foot pedalling) between the experimental and control groups over time: (a) Intention-to-treat analysis (n=673) and (b) Complete case analysis (n= 593)

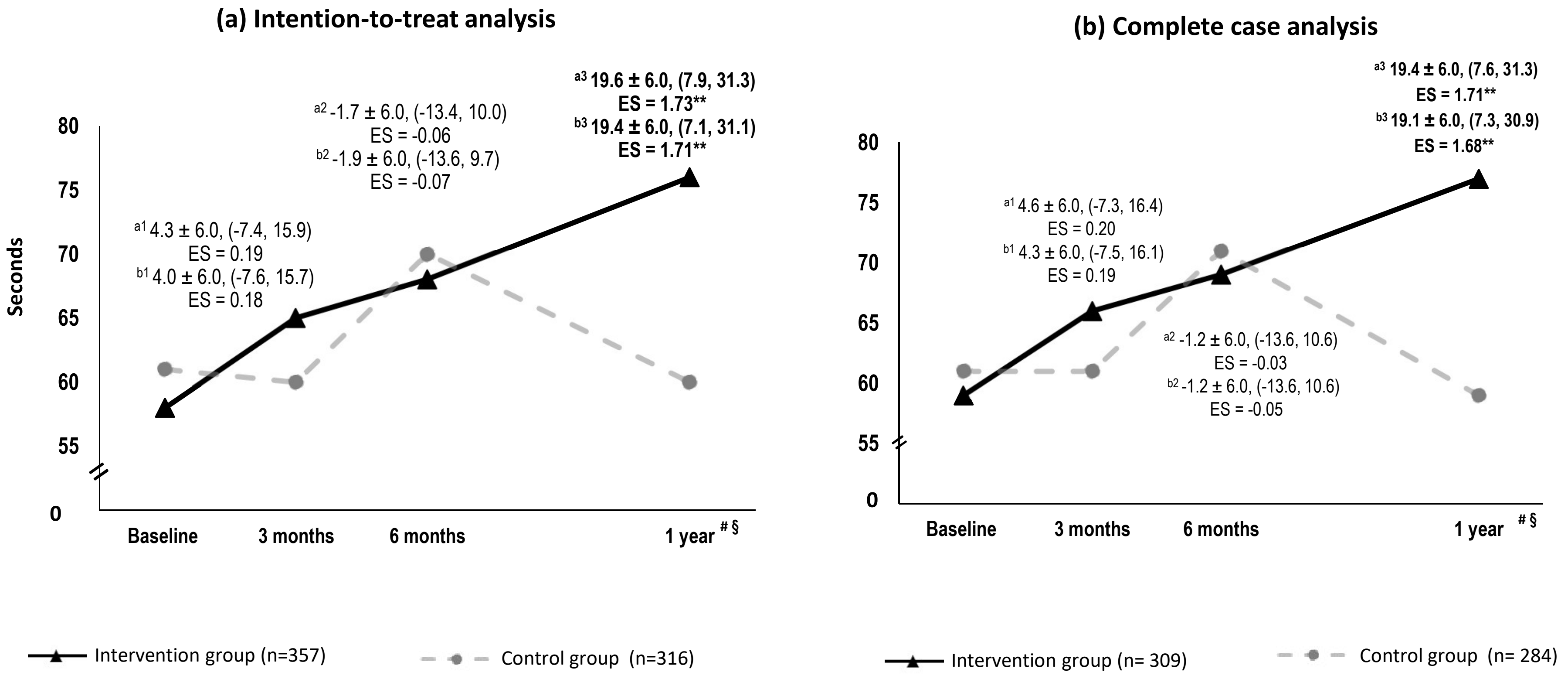

Within group change during 1-year follow-up period: #$p < 0.001$ in the experimental group and §$p < 0.001$ in the control group

Difference between the change in the experimental group and control group: mean ± standard error (95% confidence interval) at [1]3-month assessment, [2] 6-month assessment, and [3]1-year assessment

[a] Sensitivity analysis 2: Complete case analysis

[b] Sensitivity analysis 3: Complete case analysis adjusted for the potential confounders, including age, sex, martial status, education level, and monthly household income

The consistency of intervention effect over time was first examined by the group by time interaction. When evidence of group by time interaction effect was found, the intervention effects at the 3-month, 6-month, and 1-year assessment were reported separately. When no interaction effect was found, the overall intervention effect was reported.

ES = effect size; (Cohen's d): small = 0.20, medium = 0.50, large = 0.80; ‡$p < 0.1$ *$p < 0.05$, **$p < 0.01$, ***$p < 0.001$

Supplementary Fig. S3 The changes in physical activity between the experimental and control groups over time: Complete case analysis (n=593)

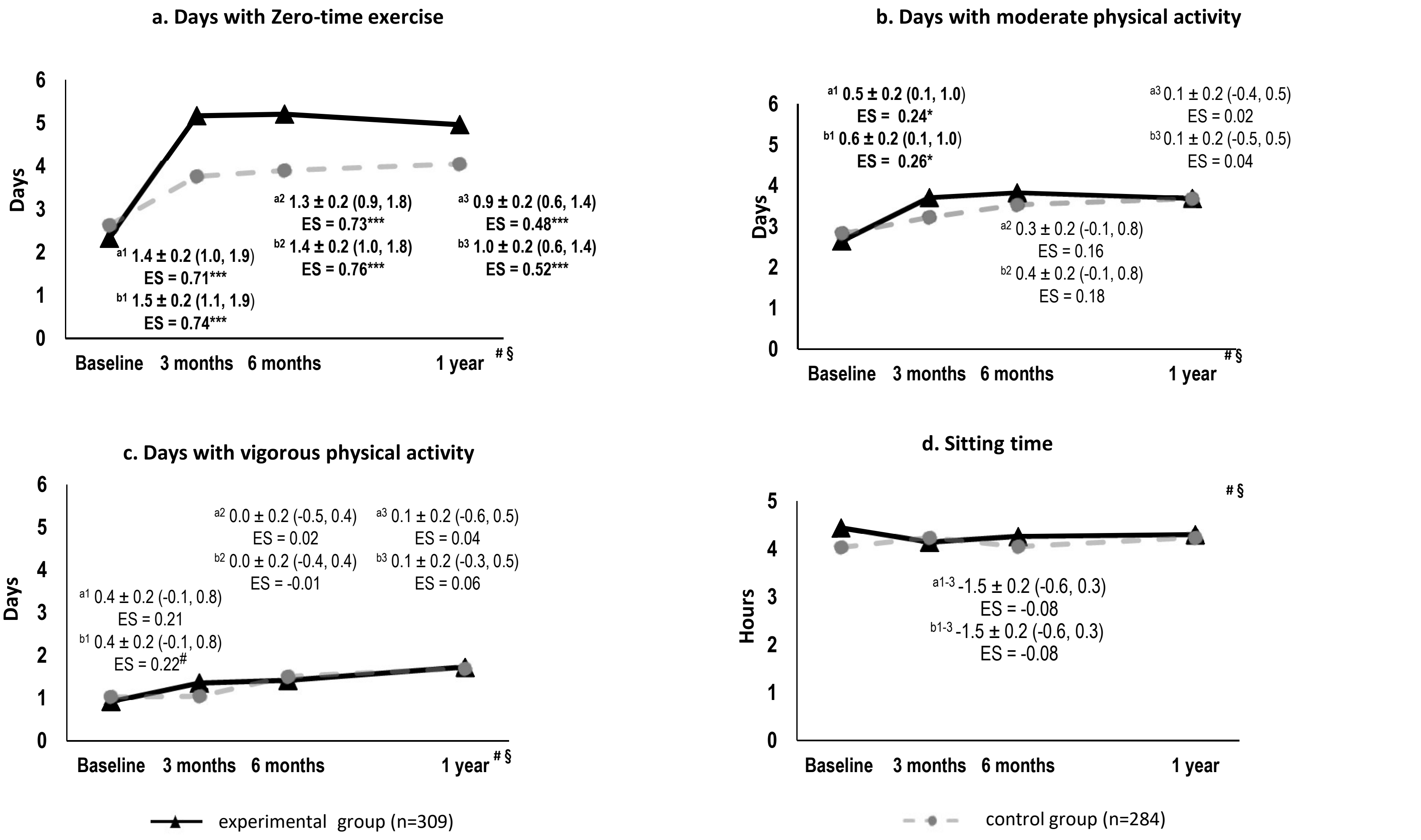

Zero-time exercise refers to simple strength-and stamina-enhancing physical activity

Within group change during 1-year follow-up period: #$p < 0.001$ in the experimental group and §$p < 0.001$ in the control group

Difference between the change in the experimental and control groups: mean ± standard error (95% confidence interval) at [1]3-month,assessment [2] 6-month assessment, and [3]1-year assessment

[a] Sensitivity analysis 2: Complete case analysis

[b] Sensitivity analysis 3: Complete case analysis adjusted for the potential confounders, including age, sex, martial status, education level, and monthly household income

The consistency of intervention effect over time was first examined by the group by time interaction. When evidence of group by time interaction effect was found, the intervention effects at the 3-month, 6-month, and 1-year assessment were reported separately. When no interaction effect was found, the overall intervention effect was reported.

ES = effect size; (Cohen's d): small = 0.20, medium = 0.50, large = 0.80; ǂ$p < 0.1$ *$p < 0.05$, **$p < 0.01$, ***$p < 0.001$

Supplementary Fig. S4 The changes in family communication and perceived well-being between the experimental and control groups over time: Complete case analysis (n=593)

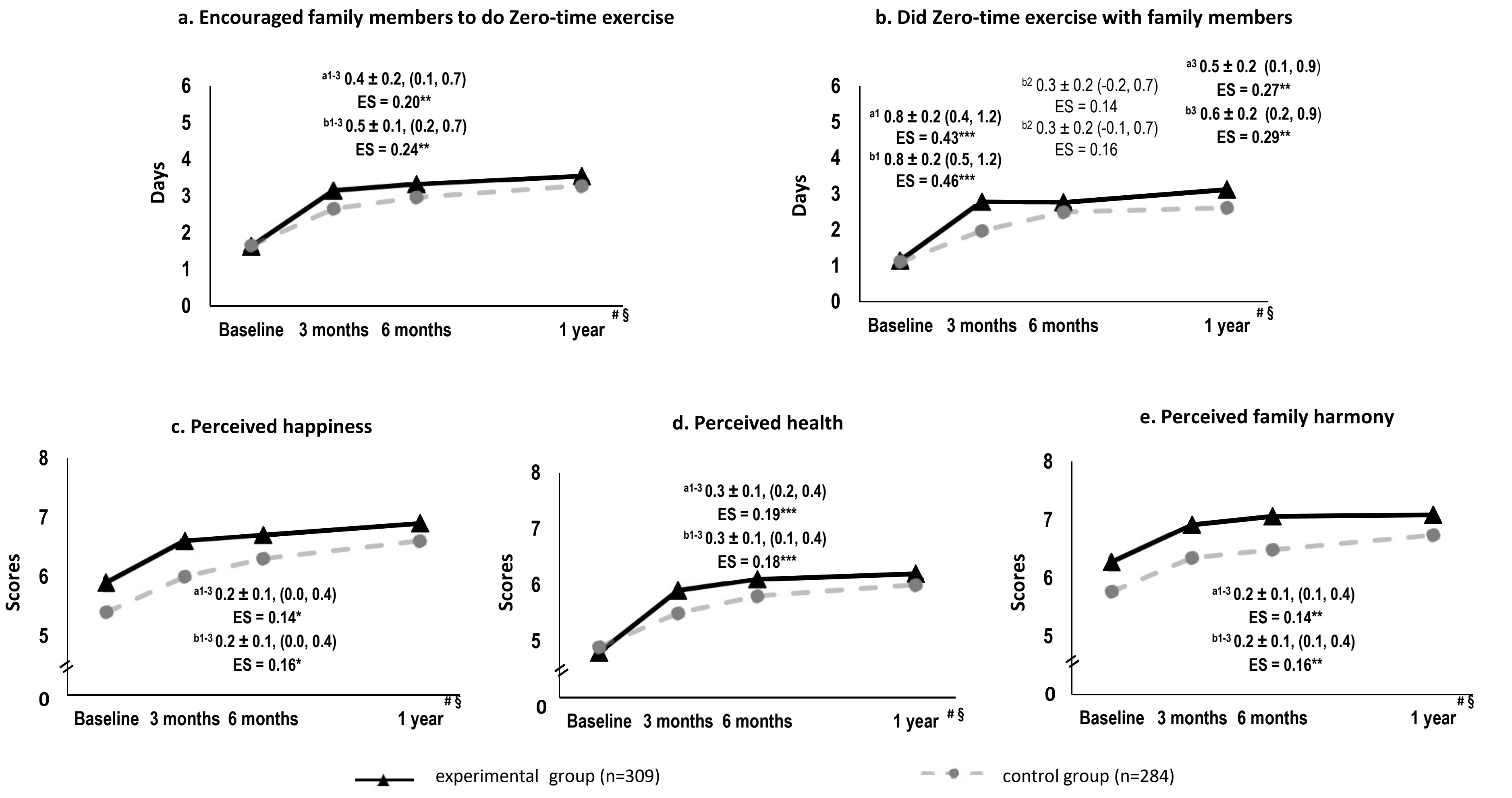

Zero-time exercise refers to brief strength-and stamina-enhancing physical activity

Within group change during 1-year follow-up period: $^{\#}p < 0.001$ in the experimental group and $^{\S}p < 0.001$ in the control group

Difference between the change in the experimental group and control group: mean ± standard error (95% confidence interval) at [1]3-month assessment, [2] 6-month assessment, and [3]1-year assessment

[a] Sensitivity analysis 2: Complete case analysis

[b] Sensitivity analysis 3: Complete case analysis adjusted for the potential confounders, including age, sex, martial status, education level, and monthly household income

The consistency of intervention effect over time was first examined by the group by time interaction. When evidence of group by time interaction effect was found, the intervention effects at the 3-month, 6-month, and 1-year assessment were reported separately. When no interaction effect was found, the overall intervention effect was reported.

ES = effect size; (Cohen's d): small = 0.20, medium = 0.50, large = 0.80; ǂ$p < 0.1$ *$p < 0.05$, **$p < 0.01$, ***$p < 0.001$
